# Physician Gender and Its Association With Patient Satisfaction and Visit Length: An Observational Study in Telemedicine

**DOI:** 10.7759/cureus.29158

**Published:** 2022-09-14

**Authors:** Kathryn A Martinez, Michael B Rothberg

**Affiliations:** 1 Center for Value Based Care Research, Cleveland Clinic, Cleveland, USA

**Keywords:** physician compensation, telemedicine, gender differences, visit length, patient satisfaction

## Abstract

Introduction

Female physicians conduct longer visits than male physicians, with negative implications for their compensation. Yet patients often report higher satisfaction with female physicians. It is unknown whether satisfaction scores for female physicians are associated with their visit lengths. Our objective was to characterize the role of the physician and patient gender with respect to visit length and patient satisfaction.

Methods

We conducted an observational cohort study with patients and physicians of a nationwide telemedicine service between 2016 and 2018. Visit length was measured by the telemedicine system. Patients rated physicians on scales of one to five stars, with five considered “top box” satisfaction. We used logistic regression to estimate differences in patient satisfaction and linear regression to estimate differences in visit length by the physician and patient gender. We tested interactions between physician and patient gender and accounted for clustering by the physician.

Results

Among 102,664 visits with 405 physicians, the mean visit length was 5.8 minutes. Visits with male physicians were 1.11 minutes shorter than those with female physicians (95% CI:-1.58, -0.65). Controlling for visit length, male physicians were less likely than female physicians to receive top-box satisfaction scores (OR: 0.72; 95% CI: 0.61, 0.85). Visits between female physicians and male patients were the longest and visits between male physicians and female patients were the shortest. Female physicians had longer visits than male physicians but this did not explain their higher satisfaction scores.

Conclusions

To reduce inequity in compensation resulting from differences in visit length, female physicians could shorten their visits without negative consequences for their satisfaction ratings.

## Introduction

Patient satisfaction metrics are often used to assess the quality of medical care and may affect physician compensation [1], patient volume, or reputation. Poor satisfaction measures can also have negative consequences for physician job satisfaction [2].

Female physicians spend more time with patients than their male colleagues [3-5]. This has important implications for physician compensation, given most physicians are compensated based on productivity. A recent national study of primary care physicians found the short additional amount of time female physicians spend with patients relative to their male counterparts directly contributes to medicine’s gender pay gap [5]. Patients report higher satisfaction with physicians who spend more time with them [6]. Yet whether this extra time results in higher satisfaction scores for female physicians is unknown. 

Findings from studies on differences in patient satisfaction by physician gender have been mixed. Most studies of real-world patient populations assess satisfaction via Press Ganey surveys. One such study in otolaryngology found no difference in patient satisfaction by physician gender [7], while another in outpatient gynecology found patients rated male physicians more highly [8]. While Press Ganey surveys are ubiquitous, they typically have extremely low response rates. They are also distributed weeks after the visit, making it harder for patients to recall specific aspects of their experience. Some versions request patients to reflect on care over the past 12 months, rather than an individual visit. As a result, understanding of differences in patient satisfaction by physician gender remains limited.

We previously found antibiotic prescription receipt was associated with shorter visit lengths [9] and higher patient satisfaction [10]. Some studies have found that female physicians are more conservative in prescribing [11-13]. No study of differences in patient satisfaction or visit length by physician gender has accounted for prescription receipt, ignoring a potentially important confounder. Whether differences in prescribing explain gender differences in visit length or patient satisfaction is unknown. 

The objective of our study was to characterize the role of physician gender with respect to patient satisfaction, controlling for prescription receipt and visit length, in a large direct-to-consumer (DTC) telemedicine service. This article was previously presented as an oral presentation at the 2022 Academy Health Annual Research Meeting on June 5, 2022.

## Materials and methods

This study uses data from the American Well DTC telemedicine platform, collected between July 2016 and July 2018. This is a nationwide telemedicine platform connecting patients and physicians 24 hours a day via synchronous video visits, accessible over smartphones, tablets, or computers. The majority of visits are for low acuity conditions, including respiratory tract infections, urinary tract infections, or otitis media [6]. Patients are connected to physicians via appropriateness and availability, yet if multiple physicians are available at a given time, patients can select among them. Patients and physicians have no prior relationship. This platform has been described in detail previously [6]. We included visits with all patients aged 18 years and over connecting with the platform for all visit reasons. Included physicians were board certified and drawn from all regions of the U.S. This study was approved by Cleveland Clinic’s Institutional Review Board.

Unlike other studies in which visit length was measured via a manual timestamp, in our study visit length was assessed automatically by the telemedicine platform. This constituted the time during which the patient and physician communicated directly via the video interface. To avoid biasing our estimates by including outlier visits in terms of length, we only included visits with a duration of 1-20 minutes.

Patient satisfaction was assessed via the telemedicine platform after each visit using a five-star rating scale, with five stars being the highest satisfaction and one star being the lowest. We dichotomized scores as 5 stars versus ≤4 stars. This scoring method is consistent with Press Ganey's “top box” scoring [14], as well as our prior work [15,16]. 

We determined whether patients received prescriptions via National Drug Codes associated with each visit and dichotomized prescription receipt as yes or no.

We coded patient and physician gender as male or female. Patient gender was self-reported and physician gender was determined via name and publicly available photo, using a method done for a prior study [15].

Statistical analysis

We generated descriptive statistics for key measures including patient gender, physician gender, visit length, patient satisfaction, and prescription outcome. We then described differences in these measures by physician gender using the chi-square statistic or analysis of variance (ANOVA). To assess differences in prescribing by physician gender, we used mixed effects logistic regression to estimate the odds of the visit resulting in a prescription, controlling for patient gender. We then used mixed effects logistic regression to estimate the association between physician gender and patient satisfaction in one model, controlling for visit length, prescription outcome, and patient gender. To assess the potential value of a visit being scored top-box to the telemedicine platform, we used logistic regression to assess whether patients who rated their initial visit in our data were more likely to have multiple visits on the platform. We estimated differences in visit length by physician gender using mixed effects linear regression, controlling for patient gender and prescription outcome. Finally, to understand whether there were differences in any of the aforementioned outcomes by physician/patient gender concordance/discordance, we ran each of the models including an interaction between physician and patient gender. All models accounted for clustering by a physician. Analyses were conducted in Stata 16.

## Results

The response rate for the satisfaction measure was 73%. The sample included 102,664 visits with 405 physicians for whom we had satisfaction data. Thirty-seven percent of visits took place between female physicians and female patients, 17% took place between female physicians and male patients, 27% took place between male physicians and female patients, and 19% took place between male physicians and male patients. Sample characteristics are presented in Table 1. Overall, the mean visit length was 5.8 minutes. Compared to visits with male physicians, visits with female physicians were longer (6.5 minutes vs. 5.1 min, p<0.001), less frequently resulted in prescriptions (76% vs 79%; p<0.001), and were more likely to result in top-box satisfaction scores (90% vs. 87%, p≤0.001). Patients who gave their initial visit a top box rating were significantly more likely to access the telemedicine platform more than once over the study period (OR: 1.40; 95% CI: 1.31, 1.48). 

**Table 1:** Sample characteristics by physician/patient gender dyad category. SD: standard deviation.

	Female physicians	Male physicians	p-value
Overall	N=214	N=191	
Mean (SD) encounter length, mins	6.5 (3.5)	5.1 (2.9)	<0.001
Visits resulting in a prescription			<0.001
No	13,255 (24)	10,067 (21)	
Yes	42,219 (76)	37,103 (79)	
Visits resulting in top-box satisfaction rating			<0.001
No	5,757 (10)	6,282 (13)	
Yes	49,717 (90)	40,908 (87)	
Patient gender			<0.001
Female	38,148 (69)	27,929 (59)	
Male	17,326 (31)	19,261 (41)	

Table 2 presents the mixed effects logistic regression of odds of a visit resulting in a prescription. Physician gender was not associated with giving a prescription. Visits with male patients were less likely to result in prescriptions (OR: 0.69; 95% CI: 0.67, 0.72). Table 3 presents interactions between patient and physician genders for all three adjusted models. There was a significant interaction between physician and patient gender in the odds of prescription receipt. Compared to visits between female physicians and female patients, those between female physicians and male patients were significantly less likely to result in a prescription (OR: 0.67; 95% CI: 0.64, 0.70). There were no differences in the odds of prescription receipt in visits between male physicians and male or female patients, compared to visits between female physicians and patients.

**Table 2:** Mixed effects logistic regression, odds of prescription receipt, by patient and physician gender.

	Odds Ratio	95% Confidence Interval
Physician gender		
Female	1.00	
Male	1.26	0.99, 1.59
Patient gender		
Female	1.00	
Male	0.69	0.67, 0.72

**Table 3:** Interactions between physician and patient gender, logistic and linear regression models. OR: Odds ratio; 95% CI: 95% Confidence interval; Est: Estimate; Ref: Reference.

	Prescription		Satisfaction		Visit length	
	OR	95% CI	OR	95% CI	Est.	95% CI
Female physician + Female patient	1.00		1.00		Ref.	
Female physician + Male patient	0.67	0.64, 0.70	1.01	0.95, 1.07	0.30	0.25, 0.34
Male physician + Female patient	1.21	0.96, 1.53	0.68	0.58, 0.80	-1.08	-1.54, -0.61
Male physician + Male patient	0.88	0.70, 1.12	0.81	0.69, 0.96	-0.85	-1.31, -0.39
			Controlling for visit duration and prescription		Controlling for prescription	

Table 4 presents the mixed effects logistic regression of patient satisfaction. Compared to visits with female physicians, visits with male physicians were significantly less likely to receive a top box rating (OR: 0.72; 95% CI: 0.61, 0.85). Compared to female patients, male patients were more likely to give their physician a top box rating (OR: 1.10; 95% CI: 1.06, 1.15). Visits that resulted in prescriptions were significantly more likely to result in a top box rating (OR: 3.50; 95% CI: 3.35, 3.65). Visit length was not associated with patient satisfaction. There were some significant interactions between patient and physician gender (Table 3). Compared to visits between female physicians and female patients, those between male physicians and female patients were significantly less likely to result in a top box rating (OR: 0.68; 95% CI: 0.58, 0.80) as were visits between male physicians and male patients (OR: 0.81; 95% CI: 0.69, 0.96). For visits with a female physician, there was no significant interaction by patient gender.

**Table 4:** Mixed effects logistic regression, odds of rating physician 5 stars versus fewer.

	Odds Ratio	95% Confidence Interval
Prescription		
No	1.00	
Yes	3.50	3.35, 3.65
Physician gender		
Female	1.00	
Male	0.72	0.61, 0.85
Patient gender		
Female	1.00	1.06, 1.15
Male	1.10	
Visit length, per minute	0.99	0.99, 1.00

Table 5 presents the mixed effects, linear regression model of visit length, by a physician and patient gender, adjusting for the prescription outcome. Compared to visits with female physicians, those with male physicians were 1.11 minutes shorter (95% CI: -1.58, -0.65). Compared to visits with female patients, those with male patients were 0.28 minutes longer (95% CI: 0.25, 0.32). Visits that resulted in prescriptions were 0.36 minutes shorter (95% CI: -0.40, -0.32). There were significant interactions between physician and patient gender (Table 3). Compared to visits between female physicians and patients, visits between female physicians and male patients were 0.30 minutes longer (95% CI: 0.25, 0.34); visits between male physicians and male patients were 0.84 minutes shorter (95% CI: -1.31, -0.39), and visits between male physicians and female patients were 1.07 minutes shorter (95% CI: -1.54, -0.61).

**Table 5:** Mixed effects linear regression model, visit length per minute.

	Estimate	95% Confidence Interval
Prescription		
No	Reference	
Yes	-0.36	-0.40, -0.32
Physician gender		
Female	Reference	
Male	-1.10	-1.56, -0.64
Patient gender		
Female	Reference	
Male	0.26	0.22, 0.30

## Discussion

In our study in a large DTC telemedicine platform, we found female physicians were more likely to receive top box satisfaction scores compared to their male colleagues. We explored several potential explanations. Female physicians spent more time with patients, but time spent was not associated with higher scores for physicians of either gender. Prescription receipt was the strongest predictor of satisfaction with physicians, but did not explain the gender discrepancy, because female and male physicians prescribed at similar rates overall. This suggests that there is something else that female physicians do that influences patients’ higher ratings of them compared to male physicians. 

The additional time that female physicians spend with patients [3,5,17] can negatively impact their revenue [5]. While visits were short overall, male physicians spent nearly 20% less time with patients compared to female physicians. Given that patients prefer for physicians to spend more time with them, we sought to understand if this additional time produced higher patient satisfaction ratings. To our knowledge, ours is the first study to test this. Physicians are largely compensated based on the number of patients they see, not on how well those patients rate them. Because ratings were not associated with visit length, it is possible that female physicians could close the gender revenue gap by spending less time with patients without causing a decline in their satisfaction metrics. That said, it’s unclear to what extent female physicians can control the length of their visits. While studies have found female physicians spend more time in patient-centered communication and building rapport with patients - both of which take time - patients might also be more inclined to disclose detail or ask questions of female physicians, resulting in a longer time expenditure. Understanding how time is used during visits with female versus male physicians is needed. 

Patient expectations for physicians can vary by physician gender [18]. One study found female physicians displayed more patient-centeredness, but this did not strongly correlate with patient satisfaction. For male physicians who practiced patient-centered communication, this was associated with higher satisfaction, suggesting gendered expectations for physician behavior and communication [19]. Indeed, another study found patient satisfaction was related to different attributes between male and female physicians. Patients liked female physicians whose communication style was in line with their gender role (e.g., soft voice, leaning into the patient), whereas male physicians’ satisfaction was less tied to gender-specific behaviors [20].

The distribution of patient visits was not uniform by physician gender. The highest concentration of visits occurred between female physicians and female patients. While patients accessing DTC telemedicine have a limited choice of physicians, it appears that female patients in our study had a preference for female physicians, a finding that has been demonstrated previously [5]. Conversely, male physicians only saw 2% more male patients than female physicians did, suggesting male patients have weaker preferences regarding their physician’s gender. We found interactions between physician and patient gender concordance. Visits between female physicians and male patients were significantly less likely to result in a prescription compared to visits between female patients and physicians. While some studies have documented more conservative prescribing by female physicians, we believe ours is the first to demonstrate that this may be influenced in part by the gender of the patient they see.

While not the primary objective of our study, we found patient gender also contributed to differences in prescription receipt, visit length, and satisfaction ratings. Male patients were less likely than female patients to receive prescriptions, yet they were more likely to be satisfied with their visit. They also had significantly longer visits than female patients, even after controlling for their lower likelihood of receiving a prescription. Studies of differences in satisfaction by patient gender have shown inconsistent results [21,22]. Ours is the first study to account for the prescription receipt and visit length in exploring this question. Our findings that male patients are happier with their DTC telemedicine experience may be the result of different expectations for care by patient gender. Women are greater healthcare consumers than men and are more likely to make healthcare decisions for other members of their family [23]. More well-developed preferences for healthcare interactions may result in a higher threshold for women to give a top-box satisfaction score to their provider.

There are some features of DTC telemedicine compared to traditional outpatient care that may affect how patients rate their care. Akin to the emergency department or urgent care, patients accessing services via DTC telemedicine have minimal choice in their physician. They also have no longitudinal relationship with the physician over which to build trust or rapport. As a result, some aspects of the visit may more strongly influence patient satisfaction than would be the case in traditional outpatient care. Indeed, several studies have found only a weak association between antibiotic receipt for respiratory tract infections and patient satisfaction in traditional outpatient care [24,25], yet in our prior studies in DTC telemedicine, we found a very strong association [10,16]. Patients may view DTC telemedicine more like a transaction and less like a typical doctor’s visit. That said, high satisfaction ratings are good for business. Giving a top-box rating to their initial visit was associated with patients having multiple visits during the study period. 

This study had some limitations. We were unable to account for patients’ stated visit reasons or their diagnoses, however, we have little reason to suspect this would have influenced the differences we found in patient satisfaction concerning gender concordance with physicians. As noted previously, we were not privy to the content of the visits and do not know whether the differences in visit length were due to physician or patient communication practices. These data are from one DTC telemedicine platform and therefore may not be generalizable to others.

## Conclusions

Addressing the gender pay gap in medicine will require a heavy emphasis on gender disparities in physician compensation by health systems. Yet volume is a major contributor to revenue at the individual physician level, and volume is directly related to time. Similar to a prior study, we found that female physicians had longer visit lengths than male physicians. Yet this did not explain their higher satisfaction scores relative to their male peers. It is therefore possible that some inequity in compensation by physician gender could be addressed through female physicians shortening their visit lengths, however, this needs to be evaluated in a prospective study. 
